# *Enterococcus faecalis* Inhibits Superantigen Toxic Shock Syndrome Toxin-1-Induced Interleukin-8 from Human Vaginal Epithelial Cells through Tetramic Acids

**DOI:** 10.1371/journal.pone.0061255

**Published:** 2013-04-22

**Authors:** Amanda J. Brosnahan, Joseph A. Merriman, Wilmara Salgado-Pabón, Bradley Ford, Patrick M. Schlievert

**Affiliations:** 1 Department of Microbiology, University of Minnesota Medical School, Minneapolis, Minnesota, United States of America; 2 Department of Microbiology, University of Iowa Carver College of Medicine, Iowa City, Iowa, United States of America; 3 Department of Pathology, University of Iowa Carver College of Medicine, Iowa City, Iowa, United States of America; Columbia University, United States of America

## Abstract

The vaginal mucosa can be colonized by many bacteria including commensal organisms and potential pathogens, such as *Staphylococcus aureus*. Some strains of *S. aureus* produce the superantigen toxic shock syndrome toxin-1, which can penetrate the vaginal epithelium to cause toxic shock syndrome. We have observed that a female was mono-colonized with *Enterococcus faecalis* vaginally as tested in aerobic culture, even upon repeated culture for six months, suggesting this organism was negatively influencing colonization by other bacteria. In recent studies, we demonstrated an “outside-in” mechanism of cytokine signaling and consequent inflammation that facilitates the ability of potential pathogens to initiate infection from mucosal surfaces. Thus, we hypothesized that this strain of *E. faecalis* may make anti-inflammatory factors which block disease progression of more pathogenic organisms. *E. faecalis* MN1 inhibited interleukin-8 production from human vaginal epithelial cells in response to the vaginal pathogens *Candida albicans, Gardnerella vaginalis*, and *Neisseria gonorrhoeae*, as well as to toxic shock syndrome toxin-1. We further demonstrated that this organism secretes two tetramic acid compounds which appear responsible for inhibition of interleukin-8 production, as well as inhibition of T cell proliferation due to toxic shock syndrome toxin-1. Microbicides that include anti-inflammatory molecules, such as these tetramic acid compounds naturally produced by *E. faecalis* MN1, may be useful in prevention of diseases that develop from vaginal infections.

## Introduction

The vaginal mucosa is a dynamic environment that consists of stratified, nonkeratinized, squamous epithelium, vaginal secretions, and commensal bacteria that reside on the surface. Most adult, healthy women are predominantly colonized vaginally by lactobacilli, but other organisms, including *Enterococcus*, coagulase-positive and negative staphylococci, *Leuconostoc, Weissella*, and *Escheichia coli*, commonly may also be found [1], [2], [3], [4], [5], [6]. Lactobacilli have multiple mechanisms to prevent the vaginal overgrowth of pathogenic microorganisms. Typically, during reproductive years, the pH of the vaginal environment at times other than menstruation is 4.0–4.5, providing an unfavorable colonization environment for many pathogens. This is primarily due to production of lactic acid by vaginal lactobacilli [7]; however other lactic acid-producing bacteria, such as *E. faecalis*, can also potentially contribute to the acidic environment. Both lactobacilli [8] and *E. faecalis* [9], [10] also produce hydrogen peroxide, which is known to be directly cytotoxic to various microorganisms. Additionally, hydrogen peroxide made by commensal organisms can act as a substrate for host-derived peroxidases to make more potent toxic compounds, or even induce host cells to make more hydrogen peroxide themselves [8], [11].

*Staphylococcus aureus* is a potentially pathogenic bacterium that colonizes up to 30% of women vaginally. Of women colonized, one-third of strains may be positive for the superantigen toxic shock syndrome toxin-1 (TSST-1) [12], [13], and thus have the ability to cause menstrual toxic shock syndrome (TSS). Although vaginal *S. aureus* usually remain localized on mucosal surfaces, TSST-1 gains access to the submucosa and bloodstream to induce massive cytokine production from macrophages and T cells, with consequent TSS. We have previously demonstrated that TSST-1 and other superantigens induce proinflammatory cytokines and chemokines from human vaginal epithelial cells (HVECs), which may act to recruit adaptive immune cells to the submucosa to initiate the cascade of events that leads to TSS [14], [15], [16]. We have referred to this as the “outside-in” signaling mechanism that counterintuitively leads to disease production, rather than protection by the activated immune system. Stated concisely, epithelial cell production of cytokines and chemokines in response to *S. aureus* or TSST-1 attracts components of the adaptive immune system that provide a pool of T cells and macrophages for disease production [14], [17]. This same mechanism has also been suggested for simian immunodeficiency virus (SIV) and HIV-1 transmission [18].

Changes in the vaginal tract microenvironment through the “outside-in” signaling mechanism may increase risk for all sexually transmitted infections [19], [20], [21]. For example, Fichorova et al. demonstrated that the spermicide nonoxynol-9 (N-9) may act to increase the risk for HIV-1 transmission [22]. N-9 induces strong inflammatory responses in the vaginal epithelium, especially after repeated exposures, which was thought to be responsible for the recruitment of HIV-1 target CD4^+^ T cells to the mucosal surface [22]. In contrast, we have recently demonstrated that an anti-inflammatory compound, glycerol monolaurate (GML), prevents transmission of SIV in a rhesus macaque model of HIV infection [18]. GML appears to inhibit epithelial cell responses by stabilizing epithelial cell membranes, thereby preventing signaling that leads to cytokine and chemokine production by the epithelial cells. We proposed that GML effectively blocks inflammatory signaling at the mucosal surface, therefore preventing the recruitment of SIV target T cells to the submucosa. Additionally, GML appears to inhibit the function of bacteria two-component systems and dissipate potential difference across membranes, resulting in pathogen killing [23].

Recently, we identified a strain of *E. faecalis* (MN1) that was isolated from a young adult healthy woman who appeared to be mono-colonized with the organism for at least 6 months. Since in our experience pure aerobic vaginal cultures are an uncommon occurrence in women, this indicated a possible ability of this organism to inhibit colonization by other microorganisms, including potential pathogens. We hypothesized that *E. faecalis* MN1 may have anti-inflammatory and properties that allow the organism to prevent co-colonization by pathogens that induce inflammation in the vaginal mucosa. Using three vaginal pathogens, as well as the superantigen TSST-1 as a model proinflammatory agent, we conducted a series of experiments to examine the ability of *E. faecalis* MN1 to inhibit the chemokine response of vaginal epithelial cells.

## Materials and Methods

### Ethics Statement

These studies were approved by the University of Minnesota Institutional Review Board and assigned protocol number 0312M54429. Written informed consent was obtained from the study participant. The study participant was a healthy young adult woman who was evaluated three times for aerobic vaginal microflora over a 6 month time period.

### Tissue culture

Immortalized HVECs from the University of Iowa, Microbiology Department have been previously described [16], [24], [25]. HVECs were maintained in Keratinocyte Serum Free Medium (KSFM; Gibco, Invitrogen, Carlsbad, CA) supplemented with bovine pituitary extract and epidermal growth factor as provided by the manufacturer, and a 1% final volume of penicillin-streptomycin (Sigma-Aldrich, St. Louis, MO) and amphotericin B (Fungizone; Gibco, Invitrogen). The cells were grown at 37°C in the presence of 7% CO_2_. On days of experimentation, antimicrobials were not used since we observed that amphotericin B reduces cytokine production by these cells. For some assays, cellular cytotoxicity was determined using the CellTiter 96® AQ_ueous_ Assay (Promega, Madison, WI).

### Microbial organisms

*E. faecalis* MN1 was isolated from a healthy woman on 3 occasions over a period of 6 months. Multiple colonies from sheep blood agar plates were evaluated at each sampling and determined to have the same phenotype, and thus were likely to be the same organisms. The organisms were shown to be gram-positive pleotropic coccobacilli that were catalase-negative, α-hemolytic on sheep blood agar plates with a distinctive pleasant odor when grown in the presence of 7% CO_2_. Despite initially being identified as *Lactobacillus* by a diagnostic microbiology laboratory, the organism was recently definitively identified as *E. faecalis* by both 16S rDNA sequencing and MALDI-TOF mass spectrometry. Briefly, for identification, 24 h cultures were grown as above, and DNA was purified from single colonies with a Qiagen EZ1 robot (Qiagen, Germantown, D). Prepared DNA (12.5 ng) was sequenced using an ABI MicroSeq 500 kit and ABI 3130 capillary sequencer per the manufacturer's instructions (both Life Technologies Corp., Grand Island, NY). The resulting sequence was processed with the IDNS SmartGene system (v.3.6.10; SmartGene GmbS, Lausanne, Switzerland) and interpreted as *E. faecalis* according to the standards published in CLSI document MM-18. In parallel, MALDI-TOF identification was performed using the Bruker BioTyper system (v.3.1, Bruker Daltonics Inc., Billerica MA) from directly-smeared colonies with a formic acid overlay, yielding a BioTyper score of 2.29 and a species identification of *E. faecalis*. The organism was maintained as a lyophilized stock culture in our laboratory.

*Gardnerella vaginalis* was obtained from the Fairview University Diagnostic Lab (Minneapolis, MN), *Candida albicans* was generously provided by Dr. Dana Davis (University of Minnesota Department of Microbiology, Minneapolis, MN), *Neisseria gonorrhoeae* was obtained from the University of Minnesota Microbiology Teaching Lab (Minneapolis, MN), and *Lactobacillus crispatus* ATCC 33197 was purchased from American Type Culture Collection (Manassas, VA).

Most bacteria and *Candida* were grown overnight in Todd-Hewitt broth (TH, Becton-Dickinson and Company, Sparks, MD) at 37°C. *L. crispatus* ATCC® 33197™ was grown in MRS broth (Becton-Dickinson and Company). *N. gonorrhoeae* were cultured on chocolate agar plates and then washed from plates with phosphate buffered saline (PBS; pH 7.2). The next day, cultured bacteria and *Candida* were concentrated by centrifugation (21,000× g for 5 min) and resuspended in PBS for use in experimentation.

### Superantigen preparation

The superantigen TSST-1 was purified as previously described [26], [27]. Briefly, TSST-1 was isolated from *S. aureus* strain RN4220 (pCE107) grown in beef heart medium [28]. The culture was treated with absolute ethanol at 4°C, the precipitate resolubilized in water, and toxin purified by isoelectric focusing. Isoelectric focusing was carried out in two phases; the first phase utilized a pH gradient of 3.5 to 10, followed by another using a pH gradient of 6 to 8. TSST-1 was identified by double immunodiffusion based on its specific reactivity with a polyclonal antibody generated against the exotoxin [29]. Purity was confirmed by SDS-PAGE, which demonstrated a single protein band at a molecular weight of 22,000 Da. Purified toxin was quantified using the BioRad protein assay (BioRad Co., Hercules, CA) with the superantigen staphylococcal enterotoxin B used for standard curve generation.

### Chemokine assays

HVECs were incubated in triplicate with *E. faecalis* MN1 (1×10^7^ CFU/well) and/or TSST-1 (100 µg/ml) for 6 h at 37°C with 7% CO_2_. At the conclusion of each experiment, the media were collected and analyzed by ELISA [16] using human Quantikine® kits (R and D Systems, Minneapolis, MN). In one additional experiment, *C. albicans* (2×10^5^ CFU/well), *G. vaginalis* (2×10^6^ CFU/well), or *N. gonorrhoeae* (2×10^6^ CFU/well) +/− *E. faecalis MN1* (8×10^6^ CFU/well) were incubated with HVECs for 6 hr at 37°C with 7% CO_2_ and analyzed by ELISA.

### Hydrogen peroxide production assay

A H_2_O_2_ colorimetric detection assay kit (Assay Designs, Ann Arbor, MI) was used to determine the amount of hydrogen peroxide produced by *E. faecalis* MN1 and *L. crispatus* ATCC 33197. Samples were collected from bacteria grown overnight in KSFM and compared to standards diluted in the same medium.

### Transwell assays

Transwell permeable supports (0.4 µm pore size, Corning Costar, Corning, NY) were used to assess the ability of a secreted factor from *E. faecalis* MN1 to inhibit HVEC production of IL-8 in response to TSST-1. HVECs were grown to confluency in 24-well plates, and transwell supports were added to wells just prior to experimentation. *E. faecalis* MN1, washed and resuspended in PBS, was added to the transwells at a final concentration of 2×10^7^ CFU and incubated for 6 h at 37°C with 7% CO_2_. Catalase enzyme (100 µg/ml, Worthington Biochemical Corp., Lakewood, NJ) was added in some conditions to degrade H_2_O_2_ produced by the bacteria.

### Lactic acid assays

Lactic acid production by *E. faecalis* MN1 or *L. crispatus* ATCC 33197 (both at 1×10^7^ CFU/well) was directly measured in tissue culture medium after a 6 h incubation with TSST-1 (100 µg/ml) on HVECs using the EnzyChrom™ L-Lactate Assay Kit (BioAssay Systems, Hayward, CA). Additionally, *E. faecalis* MN1 ± TSST-1 was incubated with HVECs, and acid production was monitored by determining the pH of the tissue culture media at 3 and 6 h. Triplicate wells were averaged for each time point and compared to a medium only control. Finally, the pH of the tissue culture media was neutralized using additional KSFM (100 µl) or 1M KOH after 3 h, and pH and IL-8 production were measured after 6 h incubation of HVECs with *E. faecalis* MN1 and TSST-1.

### *E. faecalis* MN1 supernate experiments

*E. faecalis* MN1 was grown in TH or beef heart medium overnight at 37°C with shaking. Bacterial cells were removed by centrifugation (4000× g, 15 min), and the culture supernate was filter sterilized (Corning bottle top filter, 0.22 µm cutoff). In some experiments, sterile culture supernate was added to HVECs ± TSST-1 for 6 h, and IL-8 was measured by ELISA. Additionally, culture supernate was treated with a 4× volume of 80% ethanol at 4°C. The precipitate, containing high molecular weight secreted factors, was collected by centrifugation (4000× g, 15 min) and resuspended in PBS at 10× the original concentration of the culture. Unprecipitated low molecular weight factors were concentrated by lyophilization and resuspended at 10× and 480× the original concentration in PBS. Ethanol precipitates and unprecipitated material were added to HVECs ± TSST-1 and incubated for 6 h. IL-8 production by the HVECs was measured by ELISA. Additionally, Microcon centrifugal filtration devices (Millipore, Billerica, MA) were used to isolate different molecular weight fractions from filter-sterilized *E. faecalis* MN1 supernate. Flow-through fractions were collected from the devices in order to test those molecules smaller in size than each of the filters (30 kDa, 10 kDa, and 3 kDa).

In one experiment, 480× concentrated low molecular weight *E. faecalis* MN1 secreted factors were tested for GML by GC-Mass Spectrometry at the University of Minnesota College of Pharmacy, with commercial GML as the detection standard. The lower limit of detection of GML was 0.7 µg/ml.

In an additional experiment, low molecular weight secreted factors were tested for tetramic acids by mass spectrometry; the instrument used was a Waters Q-Tof Premier. The instrument is a quadrupole time of flight instrument, housed at the High Resolution Mass Spectrometry Facility at the University of Iowa. *E. faecalis* MN1 was grown for 48 h in beef heart medium at 37°C. Cells were removed by centrifugation (10,000× g, 10 min). The culture fluid (500 ml) was filtered (0.45 µm pore size) and lyophilized. The resultant powder was extracted with 50 ml of isopropanol∶water (80∶20 ratio). The mixture was then centrifuged at 10,000× g, and the clarified supernate lyophilized. The resultant lyophilized supernate powder was assayed.

### Enzymatic treatment of *E. faecalis* MN1 supernate

Filter-sterilized supernate was subjected to enzymatic treatment as previously described [30] prior to incubation with TSST-1 (100 µg/ml) and HVECs for 6 h. Briefly, deoxyribonuclease I (DNase I, Sigma-Aldrich, 650 units/mg solid), ribonuclease A (RNase A, Sigma-Aldrich, 37 units/mg solid), or protease from *Streptomyces griseus* (Sigma-Aldrich, 5.6 units/mg solid) were added to sterile supernate at a concentration of 10 µg/100 µl and incubated for 1 h at room temperature. Enzymes were then inactivated in the following ways: DNase −75°C for 10 min, RNase – DEPC was added at a final concentration of 0.1% and incubated at room temperature overnight, protease −80°C for 15 min. The following day, 20 µl volumes were added +/− TSST-1 (100 µg/ml) to each well of HVECs, and IL-8 production was measured by ELISA 6 h later.

### Superantigenicity assay

*E. faecalis* MN1 filter-sterilized supernate and dilutions of a 480× concentrated low molecular weight secreted fraction (fraction soluble in 80% ethanol) were incubated with TSST-1 and human peripheral blood mononuclear cells (PBMCs) to assay the inhibitory effect on superantigenicity [31]. The 480× concentrated fraction was prepared after growth of the organism in beef heart medium, centrifugation and filtration to remove cells, treatment with 80% (final concentration ethanol to remove high molecular weight molecules), lyophilization of the ethanol-soluble fraction, and solubilization in water to 480× relative to the original culture fluid volume. Cellular proliferation was measured based on DNA uptake of ^3^H-thymidine. Briefly, PBMCs were isolated from heparinized (100 units/ml) human blood by Ficoll-Hypaque sedimentation. Human blood was drawn in accordance with an approved University of Minnesota IRB protocol. PBMCs were cultured in RPMI 1640 medium (Lonza, Walkersville, MD) with 2% fetal calf serum (JRH Biosciences, Inc., Lenexa, KA), 200 µM L-glutamine (Sigma-Aldrich), and 1% penicillin-streptomycin (Sigma-Aldrich). Cells were incubated for three days with TSST-1 (1 µg/well) and *E. faecalis* MN1 supernate or dilutions of the concentrated low molecular weight fraction at varying doses. Eighteen hours prior to the completion of the experiment each well received 1 µCi of ^3^H-thymidine in 20 µl of medium. Cellular DNA was collected on glass-fiber filters using a MASH II® apparatus (Microbiological Associates, Bethesda, MD). A liquid scintillation counter (model LS; Beckman Instruments, Fullerton, CA) was used to measure thymidine uptake. Data were reported as the percent of wild type stimulation, based on average counts per minute (cpm) of four replicate samples.

### Statistics

Bioassays were performed in triplicate or quadruplicate and readouts averaged for each condition. Some experiments were additionally repeated for accuracy and are identified in figure legends. Error bars represent the standard error of the mean. In some cases, Student's unpaired *t* tests or one-way ANOVAs were conducted to determine statistical significance. Means were considered to be significantly different at p≤0.05.

## Results

### *E. faecalis* MN1 inhibits the IL-8 response of HVECs to vaginal pathogens

**Figure 1:**
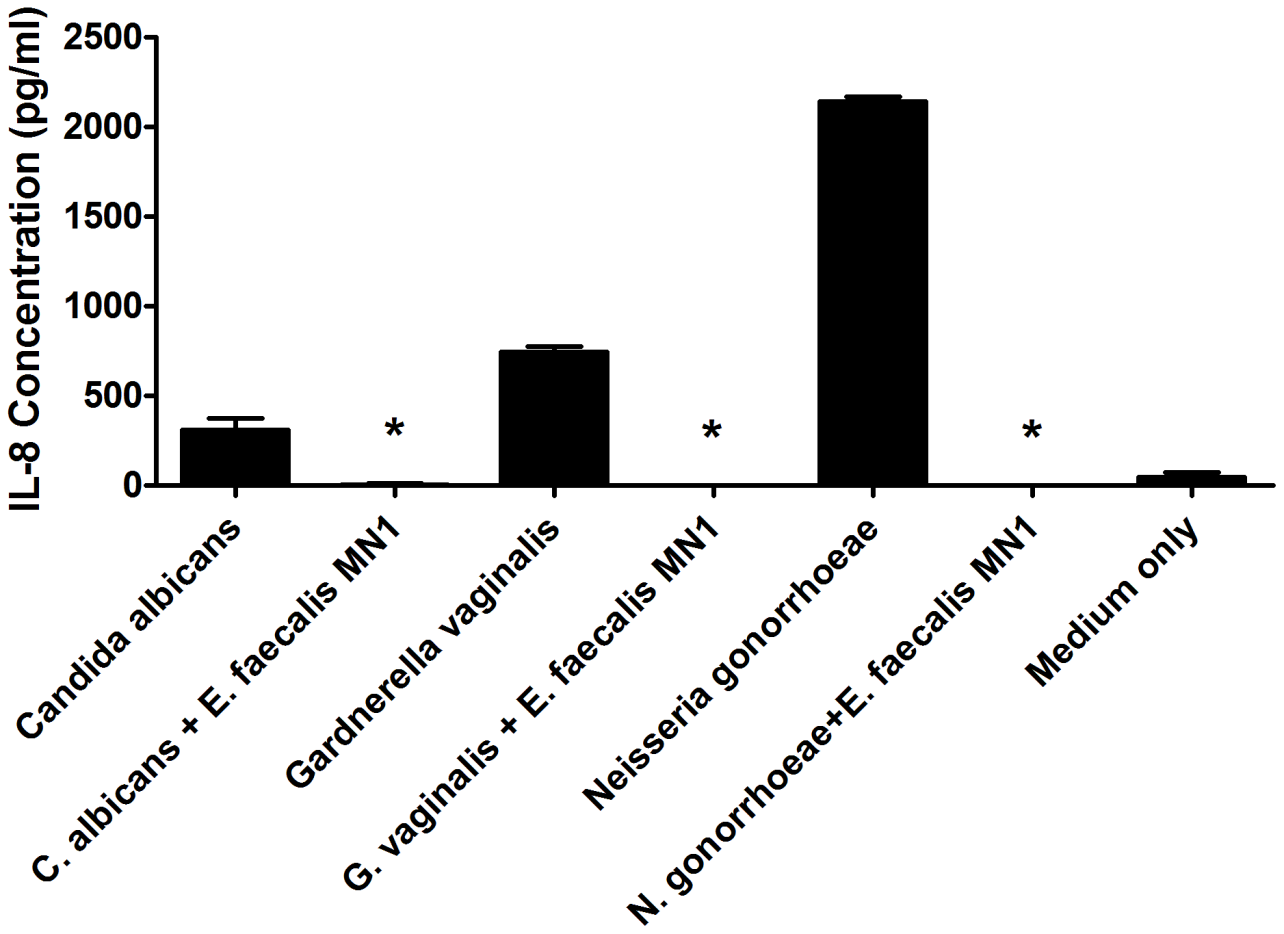
*E. faecalis* MN1 inhibits the IL-8 response of HVECs to vaginal pathogens. HVECs were incubated with *C. albicans* (2×10^5^ CFU/well), *G. vaginalis* (2×10^6^ CFU/well), or *N. gonorrhoeae* (2×10^6^ CFU/well) in the absence or presence of *E. faecalis* MN1 (8×10^6^ CFU/well) for 6 h and IL-8 production was measured by ELISA. *In each case, *E. faecalis* MN1 significantly inhibited the IL-8 response of the cells to the pathogen by individual Student's *t* tests (p<0.01). N = 3 replicates.

*E. faecalis* MN1 (8×10^6^ CFU/well) was co-cultured directly on monolayers of HVECs with either *C. albicans* (2×10^5^ CFU/well), *G. vaginalis* (2×10^6^ CFU/well), or *N. gonorrhoeae* (2×10^6^ CFU/well) for 6 h, and IL-8 production by the cells was compared to that induced by each pathogen alone. In each case, *E. faecalis* MN1 abolished the IL-8 response of HVECs to the pathogen (Fig. 1).

### *E. faecalis* MN1 inhibits the IL-8 response of HVECs to TSST-1

**Figure 2:**
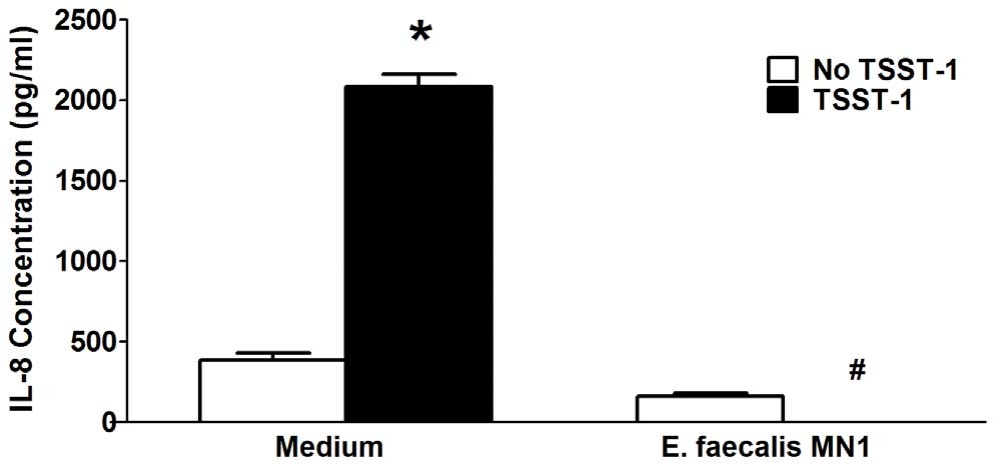
*E. faecalis* MN1 inhibits the IL-8 response of HVECs to the staphylococcal superantigen TSST-1. *E. faecalis* MN1 (1×10^7^ CFU) was incubated with HVECs in the absence and presence of TSST-1 (100 µg/ml) for 6 h. Cell culture supernates were collected and IL-8 was measured by ELISA. *Significantly higher than all other conditions and #significantly lower than medium only control by one-way ANOVA [*F*(3,29) = 251.2, *p*<0.0001] and Tukey's post-hoc test. N = 3–6 replicates.

*E. faecalis* MN1 (1×10^7^ CFU/well) was incubated directly on monolayers of HVECs in the absence or presence of TSST-1 (100 µg/ml) for 6 h, and IL-8 was then measured by ELISA. We have previously shown that the staphylococcal superantigen TSST-1 stimulates production of several cytokines and chemokines by HVECs, with IL-8 production being the most up-regulated [15], [16]; thus, we utilized the toxin for all subsequent experiments. Fig. 2 shows that *E. faecalis* MN1 completely inhibited IL-8 production by HVECs in response to the toxin. No cellular cytotoxicity was noted.

### *E. faecalis* MN1 secretes a factor responsible for the inhibition of IL-8 due to TSST-1

**Figure 3:**
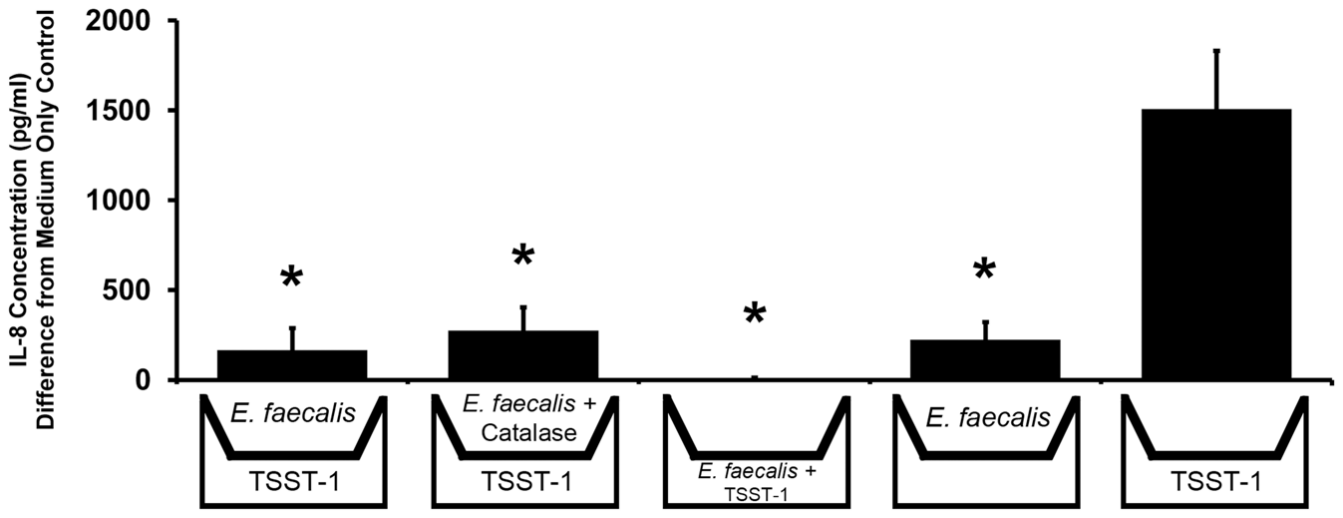
*E. faecalis* MN1 secretes a factor responsible for inhibition of IL-8 production by HVECs in response to TSST-1. Transwell permeable supports (0.4 µm pore size) were used to determine the ability of *E. faecalis* MN1 secreted factors to inhibit TSST-1-induced IL-8 production by HVECs. HVECs were grown to confluency in the bottom wells and transwell permeable supports were added on the day of experimentation. *E. faecalis* MN1 (2×10^7^ CFU) was added to either the transwell (to assess secreted factors) or the bottom well (as a positive control) in the presence or absence of TSST-1 (100 µg/ml) and incubated for 6 h. In one condition, catalase enzyme (100 µg/ml) was added to break down hydrogen peroxide produced by the lactobacilli. Cell culture supernates were collected and analyzed by ELISA for IL-8 production. These results contain data averaged from three separate experiments, with 6 total replicates in each experimental condition. *All conditions with *E. faecalis* MN1 present demonstrated significantly lower IL-8 levels compared to the TSST-1 only control by individual Student's *t* tests (p<0.01).

In order to determine if inhibition of HVEC IL-8 production by *E. faecalis* MN1 was due to a secreted factor made by the bacteria, transwell permeable supports were used to place enterococci in the tissue culture medium without allowing the bacteria to come in contact with the cells. HVECs were grown to confluency in the bottom wells of a tissue culture plate, and transwells (0.4 µm pore size) were added on the day of experimentation. As a positive control, *E. faecalis* MN1 (1×10^7^ CFU/well) was added to the lower chamber with TSST-1 (100 µg/ml) to demonstrate inhibition of IL-8 as previously noted in Fig. 2. In all other conditions, *E. faecalis* MN1 (1×10^7^ CFU/well) was added only to the upper chamber (in the transwells), and TSST-1 (100 µg/ml) was added to the bottom chamber. When *E. faecalis* MN1 was added to the transwell above the cells in the presence of TSST-1, there was significant inhibition of the IL-8 response to the toxin (p<0.01) (Fig. 3). Therefore, *E. faecalis* MN1 secretes a factor capable of migrating across a permeable membrane with a 0.4 µm diameter pore size that is responsible for inhibiting the induction of IL-8 from HVECs exposed to TSST-1. This effect was also seen when catalase (100 µg/ml) was present to degrade hydrogen peroxide made by the bacteria. In one experiment, TSST-1 was added to the upper chamber with *E. faecalis* MN1, and inhibition was similar to all other conditions (data not shown). Samples were taken from the upper and lower chambers after 6 h and plated on TH agar plates to look for enterococci contamination of the lower chambers. No enterococci were detected in the lower chambers (except for the positive control in which enterococci were added directly to the lower chamber). *E. faecalis* MN1 also remained viable in the upper chambers in all conditions after 6 h.

### Hydrogen peroxide production by *E. faecalis* MN1 does not correlate with inhibition of IL-8

**Figure 4:**
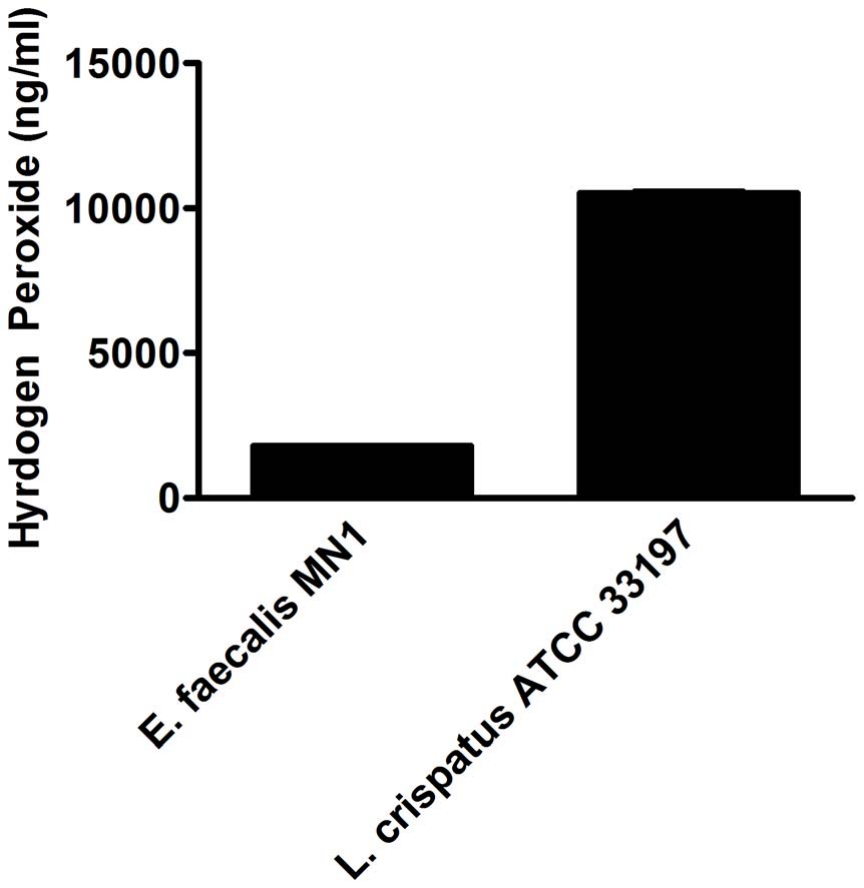
*E. faecalis* MN1 makes low levels of hydrogen peroxide. *E. faecalis* MN1 and *L. crispatus* ATCC 33197 were grown overnight in KSFM tissue culture medium. Hydrogen peroxide production was measured using a H_2_O_2_ colorimetric detection assay kit.

Two secreted factors of enterococci, hydrogen peroxide and lactic acid, have been shown to be important for growth inhibition of pathogens. Although we demonstrated that the presence of catalase did not affect HVEC IL-8 inhibition by *E. faecalis* MN1, we measured levels of hydrogen peroxide produced by the strain to confirm that this molecule did not play a role in HVEC IL-8 inhibition. *E. faecalis* MN1 and *L. crispatus* ATCC 33197 were grown overnight in KSFM tissue culture medium and assayed for hydrogen peroxide production using a colorimetric detection kit. Only low levels of hydrogen peroxide (1.78 µg/ml±3.4) were detected from *E. faecalis* MN1, compared to hydrogen peroxide production by *L. crispatus* ATCC 33197 (Fig. 4).

### Lactic acid does not solely contribute to inhibition of IL-8

**Figure 5:**
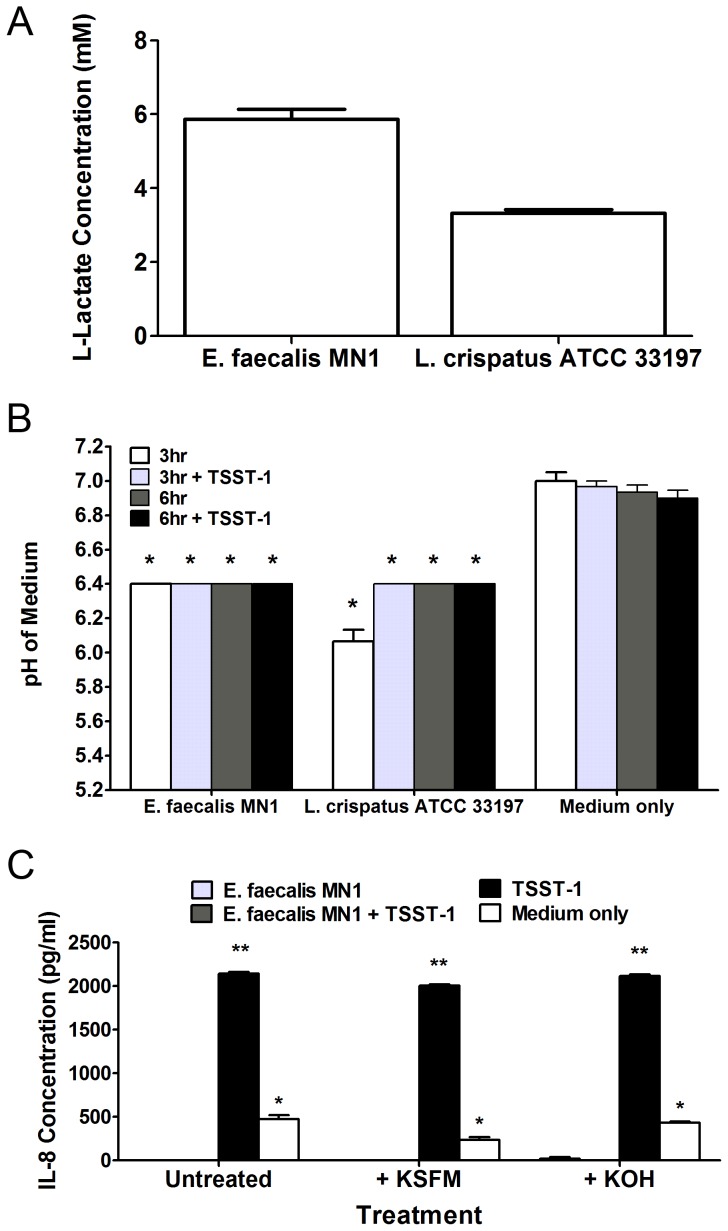
Lactic acid is not solely responsible for IL-8 inhibition. A) *E. faecalis* MN1 and *L. crispatus* ATCC 33197 (both at 1×10^7^ CFU/well) were incubated with TSST-1 (100 µg/ml) on HVECs for 6 h, and lactic acid was measured in the tissue culture medium using a colorimetric assay. B) *E. faecalis* MN1 and *L. crispatus* ATCC 33197 (both at 1×10^7^ CFU) were incubated ± TSST-1 (100 µg/ml) with HVECs for 6 h, and the pH of the tissue culture media was measured after 3 and 6 h. All conditions with enterococci or lactobacilli were significantly lower than the TSST-1 or medium only controls at 3 h and 6 h by individual Student's *t* tests (p<0.001). C) Cells were incubated with *E. faecalis* MN1 (1×10^7^ CFU) ± TSST-1 (100 µg/ml) for 6 h. After 3 h, cells were either left untreated or were treated with additional KSFM tissue culture medium or 1M KOH to neutralize the pH of the medium. IL-8 was measured after 6 h, at which point the pH of the medium in treated samples was still at or near neutral. All conditions that contained *E. faecalis* MN1, no matter the pH level, showed significantly lower levels of IL-8 than that of TSST-1 alone. **Significantly higher than all other conditions and *significantly higher than conditions containing *E. faecalis* MN1 irrespective of neutralization treatment by one-way ANOVA [*F*(3,8) = 555.1, *p*<0.0001] and Tukey's post-hoc test. N = 3 replicates for each experiment.

To determine the effect of lactic acid produced by *E. faecalis* MN1 on the inhibition of HVEC production of IL-8, we performed the following experiments. First, we measured lactic acid production by *E. faecalis* MN1 (1×10^7^ CFU/well) or *L. crispatus* ATCC 33197 (1×10^7^ CFU/well) after 6 h incubation with TSST-1 (100 µg/ml) using a colorimetric determination kit, which confirmed that *E. faecalis* MN1 makes lactic acid (approximately 6 mM) in this system (Fig. 5A). Second, the pH levels of the tissue culture medium when *E. faecalis* MN1 was incubated with HVECs in the absence or presence of TSST-1 were measured. The production of lactic acid by enterococci should cause the pH of the medium to drop over time. The pH of the tissue culture medium was measured after 3 and 6 h incubations. After just 3 h, *E. faecalis* MN1 induced significantly lower pH levels compared to medium only or TSST-1 only controls (p<0.001), and this effect was maintained after 6 h (Fig. 5B). Additionally, we neutralized the pH of the tissue culture medium after 3 h and then measured IL-8 production by the HVECs in response to TSST-1. After 3 h, the pH was measured and was 6.5 compared to medium only and TSST-1 only controls at 7.1. Addition of fresh KSFM tissue culture medium or 1M KOH to the wells brought the pH up to 7.0. After 6 h, the pH of all conditions that had received the neutralization treatment was 6.8 (controls were at 7.0). Untreated conditions containing *E. faecalis* MN1 had pHs of 6.4 after 6 h. Fig. 5C shows that in both cases, raising the pH did not counteract the ability of *E. faecalis* MN1 to inhibit IL-8 production.

### Ethanol precipitation of *E. faecalis* MN1 supernate factors shows that at least a low molecular weight factor plays a role in IL-8 inhibition

**Figure 6:**
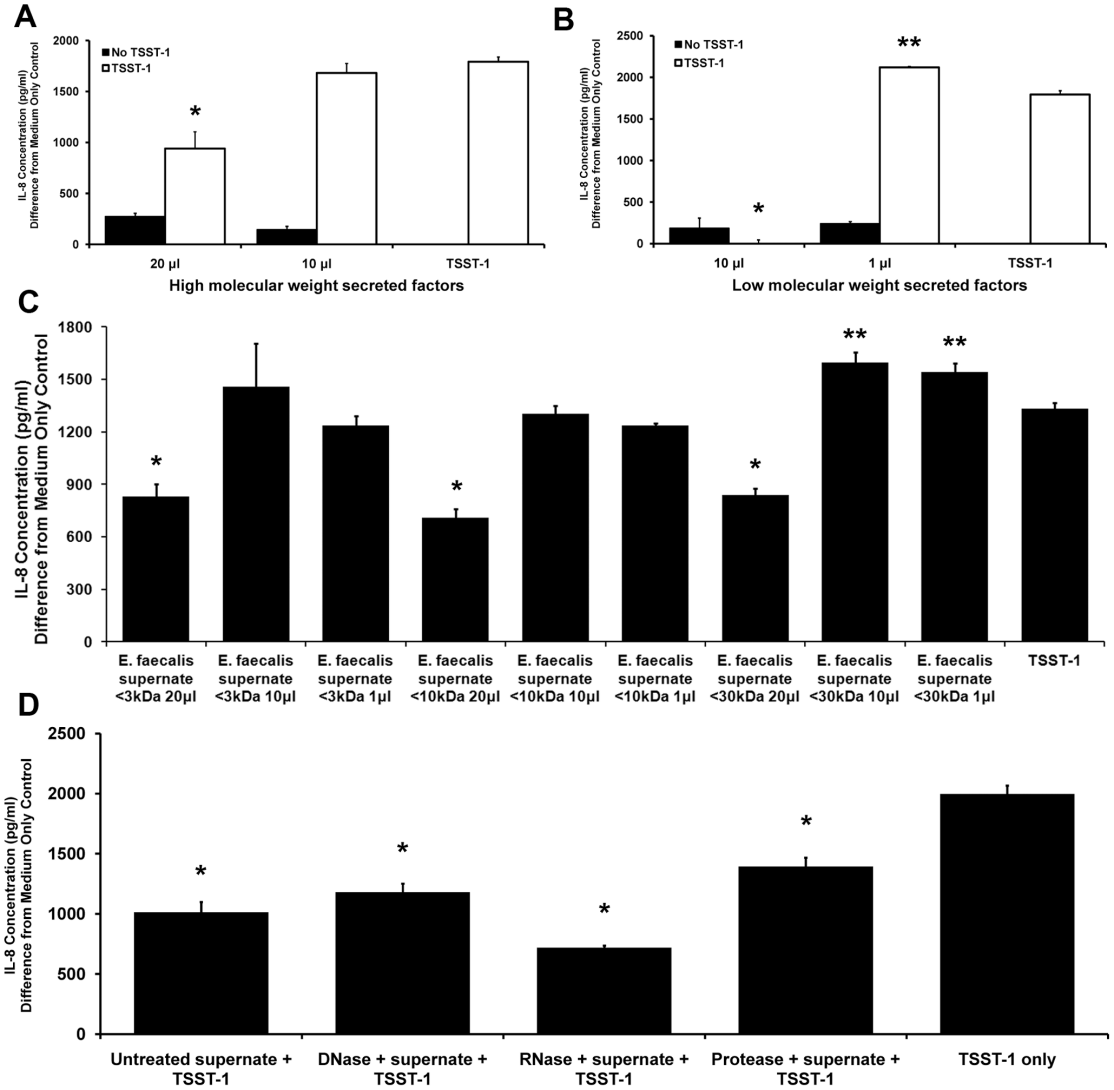
*E. faecalis* MN1 secretes a small molecule responsible for inhibition of TSST-1-induced IL-8 production by HVECs. A) Eighty percent ethanol was used to precipitate high molecular weight factors from filter-sterilized *E. faecalis* MN1 supernate. Isolated factors were added to HVECs ± TSST-1 (100 µg/ml) for 6 h, and IL-8 was measured by ELISA. Only the 20 µl condition showed inhibition of TSST-1-induced IL-8 by Student's *t* test (p<0.001). B) Low molecular weight (unprecipitated) factors were concentrated by lyophilization, resuspended in PBS to the same concentration as the precipitated material, and incubated with cells as described above. Lower doses of unprecipitated material were used to minimize cytotoxicity to the HVECs. The 10 µl condition showed absolute inhibition of IL-8 production (*p<0.001), whereas the 1 µl condition actually enhanced IL-8 levels in response to TSST-1 (**p<0.001) by individual Student's *t* tests. These results are based on the average of two separate experiments, each containing three replicates per condition. C) Filter-sterilized *E. faecalis* MN1 supernate was separated into different molecular weight fractions using Microcon centrifugal filters and incubated with HVECs +/− TSST-1 (100 µg/ml) for 6 h. Inhibition of TSST-1-induced IL-8 production is maintained in all fractions (<3 kDa, <10 kDa, <30 kDa) when the cells were incubated with 20 µl of material (*p<0.005); a higher level of IL-8 was detected when cells were incubated with smaller amounts of the <30 kDa fraction (**p<0.05) by individual Student's *t* tests. These data are from a single experiment carried out in triplicate. D) Filter-sterilized *E. faecalis* MN1 supernate was treated with DNase, RNase, or protease prior to incubation with TSST-1 (100 µg/ml) on HVECs for 6 h. None of the treatments affected the ability of the secreted factor to inhibit TSST-1-induced IL-8 production from the cells by individual Student's *t* tests (*p<0.001). These data are the average of two experiments, each done in triplicate.

Due to the fact that neither hydrogen peroxide nor lactic acid appears to be responsible for the inhibition of HVEC IL-8 production in response to TSST-1, we sought to characterize the secreted factor responsible for this effect. *E. faecalis* MN1 was grown overnight in beef heart medium, and culture supernate was collected and sterilized. Culture supernate alone inhibited the IL-8 response of HVECs to TSST-1 after 6 h (data not shown), similar to that seen with the transwell experiment (Fig. 3). Ethanol (80% solution) was used to precipitate the high molecular weight factors from the culture supernate. Additionally, unprecipitated material was collected and concentrated by lyophilization and then resuspended at the same concentration as the precipitated material. Both high and low molecular weight molecular weight fractions inhibited IL-8 production by HVECs (Fig. 6). High doses (50 µl per well) of either high or low molecular weight factors were cytotoxic to HVECs, along with a lower dose (20 µl per well) of the low molecular weight factors (data not shown). Fig. 6A shows that high molecular weight secreted factors inhibited TSST-1-induced IL-8 when 20 µl were added to the wells (p<0.001), however this effect was lost when only 10 µl of material were added. Fig. 6B demonstrates that low molecular weight secreted material had greater inhibitory activity in that it led to total prevention of IL-8 production from HVECs exposed to TSST-1 at only 10 µl (p<0.001). Inhibitory activity was lost when 1 µl of the low molecular weight material was added to the cells, and in fact the response to TSST-1 was enhanced under these conditions (p<0.001).

### Separation of *E. faecalis* MN1 supernate by molecular mass indicates that the secreted factor may be less than 3 kDa

Microcon centrifugal filter devices were used to separate fractions by molecular mass from filter-sterilized *E. faecalis* MN1 supernate. The filters used had molecular mass cutoffs of 30 kDa, 10 kDa, and 3 kDa, and flow-through material was collected to obtain all molecules smaller than those cutoffs. Fig. 6C demonstrates that 20 µl of material from any fraction was sufficient to inhibit TSST-1-induced IL-8 responses from HVECs (p<0.005). Lower amounts of the highest molecular weight fraction (<30 kDa) induced increased levels of IL-8 from the cells (p<0.05).

### Treatment of *E. faecalis* MN1 supernate with DNase, RNase, or protease does not affect the inhibitory activity

Filter-sterilized *E. faecalis* MN1 supernate was treated with DNase, RNase, or protease prior to incubation with TSST-1 and HVECs in order to determine whether the inhibitory factor was composed of DNA, RNA, or protein, respectively. Fig. 6D shows that the ability of *E. faecalis* MN1 supernate to inhibit the IL-8 response to TSST-1 was maintained in all conditions (p<0.001), indicating that the inhibitory factor was not DNA-, RNA-, or protein-based.

### *E. faecalis* MN1 supernate and a low molecular weight fraction inhibit TSST-1-induced proliferation of PBMCs

**Figure 7:**
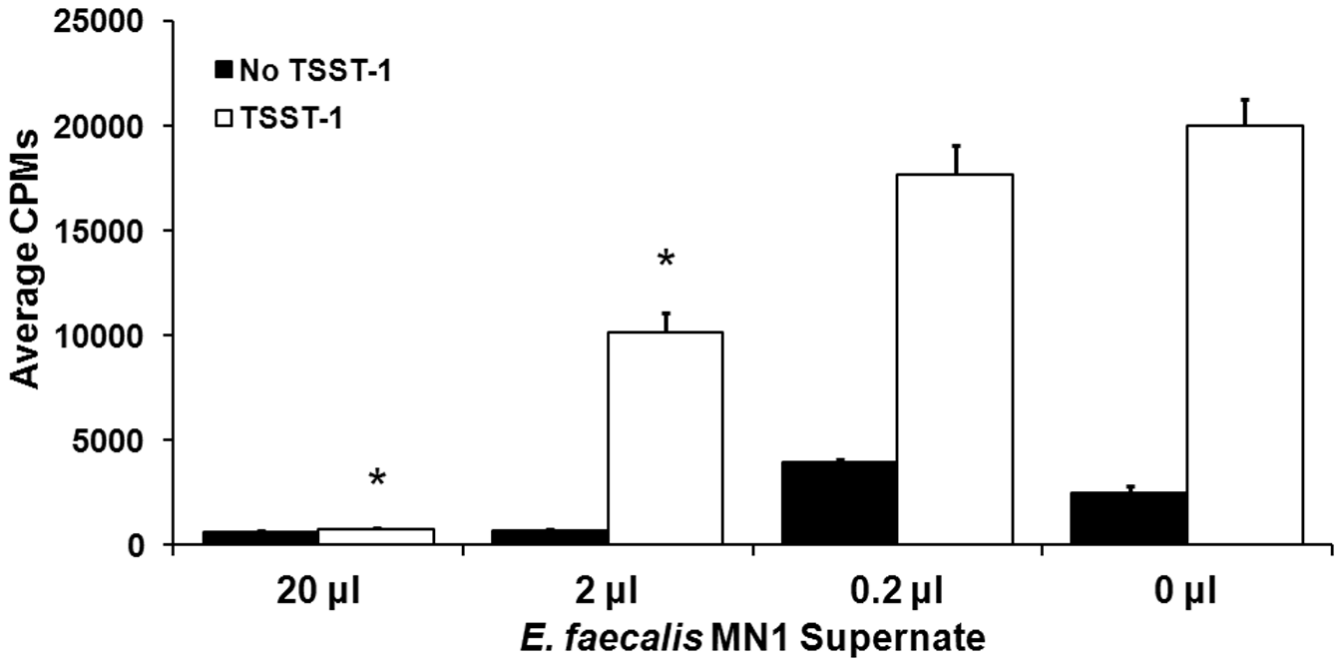
*E. faecalis* MN1 supernate inhibits TSST-1-induced T cell proliferation. *E. faecalis* MN1 supernate was incubated with human PBMCs and TSST-1 (1 µg/well) in a superantigenicity assay to assess the ability of secreted factors to inhibit TSST-1-induced T cell proliferation. The two highest doses of supernate (20 and 2 µl per well) significantly inhibited T cell proliferation due to TSST-1 by individual Student's *t* tests (*p<0.001). The 0 µl control shows the average CPMs for PBMCs with and without TSST-1.

**Figure 8:**
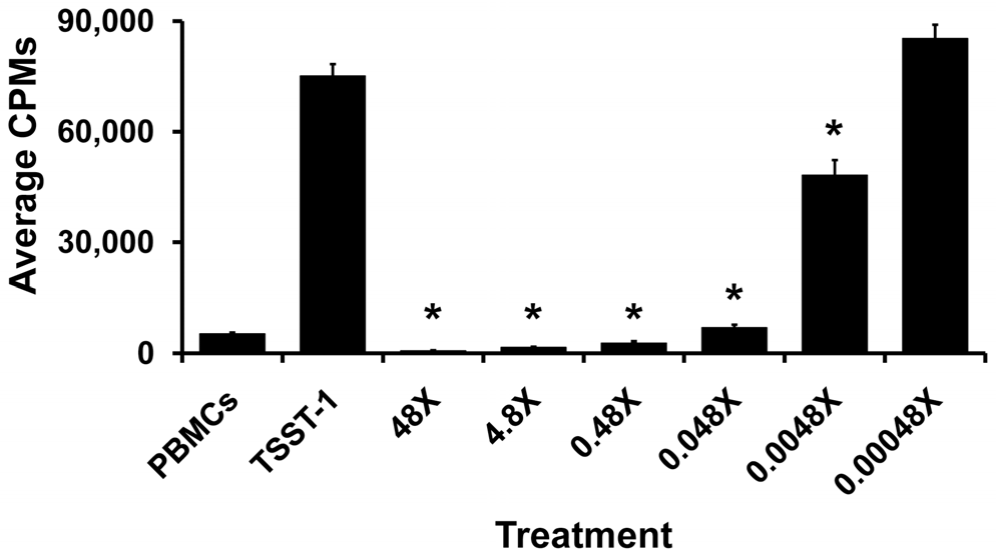
*E. faecalis* MN1 low molecular weight fraction inhibits TSST-1-induced T cell proliferation. *E. faecalis* MN1 supernate was treated with ethanol (80% final concentration), insoluble material was removed by centrifugation, soluble material (referred to as the low molecular weight fraction) was concentrated 480 times relative to the original culture fluid, and dilutions incubated with human PBMCs and TSST-1 (1 µg/well) in a superantigenicity assay to assess the ability of secreted factors to inhibit TSST-1-induced T cell proliferation. The PBMC control shows the average CPMs for PBMCs alone and TSST-1 shows the average CPMs for TSST-1 alone. Treatments 48× to 0.0048× were significantly different from TSST-1 alone by individual Student's *t* tests (*p<0.001). Three independent wells for each treatment were assayed for cell viability, and viability in all cases was >95%.

*E. faecalis* MN1 supernate was incubated with TSST-1 and human PBMCs to test the ability of the inhibitory factor to inhibit TSST-1-induced T cell proliferation (Fig. 7). *E. faecalis* MN1 supernate was capable of inhibiting T cell proliferation at two of the three doses (20 and 2 µl per well, p<0.001) when compared to the TSST-1 only control. After growth of *E. faecalis* MN1 in beef heart medium, higher molecular weight material was removed by precipitation with 80% final concentration ethanol. The low molecular weight, ethanol soluble fraction was lyophilized and concentrated 480× relative to the original culture. Dilutions of the fraction (48× to 0.00048×) were evaluated for effects on TSST-1-induced proliferation of human PBMCs (Fig. 8). For all dilutions of the low molecular weight fractions, 20 µl were added to wells containing PBMCs. All dilutions of low molecular weight fractions, except 0.00048×, significantly inhibited TSST-1-induced proliferation of PBMCs. Dilutions from 48× to 0.48× reduced the TSST-1-induced proliferation below that of PBMCs only. For all treatments, PBMC viability remained above 95% during the 4 day incubation, indicating the inhibitory factor was not cytocidal. The same dilutions of low molecular weight fractions inhibited IL-8 production by HVECs, without causing loss of HVEC viability (data not shown).

### Characterization of the *E. faecalis* MN1 low molecular weight factor

The properties of the low molecular weight factor produced by *E. faecalis* MN1 share similarities to that seen with the compound GML [23], raising the possibility that the factor was GML or a related molecule. We tested the 480× concentrated supernate for GML by GC-mass spectrometry. The concentrated supernate was negative for detectable GML. We next tested a different sample of low molecular weight extract of the culture supernate for the presence of tetramic acids, molecules which share many properties with GML, including broad antimicrobial activity and anti-inflammatory properties [32], [33]. Mass spectrometry demonstrated the presence of two tetramic acids, reutericyclin and a related tetramic acid, molecules that are likely to account for the observed activity.

## Discussion

The vaginal epithelium plays an important role in the initial response to microorganisms found in the vaginal tract. The ability of a vaginally-isolated strain of *E. faecalis* to inhibit TSST-1-induced IL-8 from the vaginal epithelium may prove to be significant since we have previously shown that “outside-in” signaling resulting in superantigen-induced inflammation may play a role in the development of TSS from the vaginal mucosa [14], [15], [17]. We have shown that TSST-1-induced inflammation initiated by epithelial cell production of cytokines and chemokines may open the mucosal barrier and recruit adaptive immune cells to the submucosa so that the superantigen can interact with these cells in order to initiate the cascade of events that leads to systemic TSS [14]. This is a similar paradigm to that proposed for HIV: the virus signals through vaginal epithelial cell production of cytokines/chemokines to recruit its target cells to the submucosa in order to initiate an active infection [18]. This “outside-in” signaling may prove to be even more important as we continue to characterize how other mucosal pathogens initiate systemic infections. Indeed, three vaginal pathogens initially examined here, *C. albicans, G. vaginalis*, and *N. gonorrhoeae*, all induced IL-8 responses from HVECs, but those responses were blocked in the presence of *E. faecalis* MN1. Therefore, these anti-inflammatory tetramic acid compounds, which can be found naturally produced by microbes in the human vaginal tract, may be useful as therapeutics to block the development of TSS and other systemic infections that start at the vaginal surface.

Lactic acid is a secreted factor that is known to interfere with colonization by potential vaginal pathogens. Although the pH of the tissue culture medium was significantly lower when *E. faecalis* MN1 was incubated with the HVECs after 3 h, neutralization of the pH at 3 h did not interfere with IL-8 inhibition. This neutralization lasted until the end of the experiment (3 h later), indicating that lactic acid does not play a role in IL-8 inhibition. It is possible, however, that a decrease in pH due to lactic acid changes the ability of the HVECs to respond to TSST-1 and that this happens rapidly after the addition of enterococci to the medium, so that neutralization after 3 h does not matter.

Our studies with *E. faecalis* MN1 show for the first time that, in addition to lactic acid, the organism produces two tetramic acid molecules, at least one highly similar to reutericyclin, which was originally isolated from *Lactobacillus reuteri* from a sourdough bread culture [34]. Reutericyclin has been shown to act as a proton-ionophore to disrupt the pH gradient of bacterial membranes, causing growth inhibition of Gram-positive organisms (other lactobacilli, staphylococci, and enterococci). Gram-negative organisms appear to be protected due to their lipopolysaccharide outer membranes; however mutation of the outer membrane confers susceptibility to reutericyclin [35], [36], [37]. Reutericyclin is also anti-inflammatory [32], [33]. Recently, we postulated that another anti-inflammatory compound, GML, acted to inhibit Gram-positive bacterial growth in a mechanism similar to that of tetramic acids [23]. Indeed, GML has structural similarities to tetramic acids, and the fatty acid monoester appears to function as a quorum sensing molecule for *Pseudomonas aeruginosa*, another organism that produces tetramic acids and is resistant to GML [23]. Interestingly, we have shown that GML also inhibits cytokine and chemokine responses from human vaginal epithelial cells in response to TSST-1 and HIV-1 [18], [38]. Thus, we propose that the two tetramic acid, reutericyclin-like compounds made by *E. faecalis* MN1 exhibit anti-inflammatory properties on vaginal epithelial cells through their potential ability to alter signal transduction across eukaryotic membranes in addition to bacterial membranes.

*E. faecalis* MN1 filter-sterilized culture supernates and low molecular weight fractions significantly inhibited T cell proliferation caused by TSST-1, likely due to the presence of tetramic acids. This effect is also observed with GML [39]. It is interesting to note that the highest concentrations of culture supernates also prevented proliferation of PBMCs in the absence of TSST-1, and the highest concentrations of low molecular weight fractions reduced PBMC proliferation below the cells only control. This confirms that the tetramic acid, reutericyclin-like compounds have a more generalized immunomodulatory effect, and their actions are not restricted to epithelial cells. These findings are also significant because they suggest that T cells recruited into the submucosa may be inhibited from proliferation due to TSST-1 or other factors such as HIV. It is well known that activated T cells are more easily infected by HIV than unactivated cells. Thus, tetramic acids (and GML) may help prevent infections in three ways: 1) antimicrobial activity; 2) inhibition of epithelial cell production of cytokines and chemokines; and 3) inhibition of activation of T cells.

The concept of reducing innate immune responses to vaginal pathogens in order to prevent the progression of disease may be counterintuitive, but in many cases too much immune system activation can be detrimental to the host. An exuberant immune response can lead to tissue destruction, which contributes to a pathogen's ability to invade deeper host tissues; alternatively, what should be a protective immune response can lead to the recruitment of host cells that can subsequently be infected by some pathogens. A balance may be necessary in the vaginal microenvironment that allows the host to respond appropriately to harmful pathogens without contributing to the progression of disease, and anti-inflammatory compounds such as these naturally secreted reutericyclin-like, tetramic acid compounds may confer unique benefits in establishing a healthy microbial equilibrium.
